# *Ziziphus jujuba*: Applications in the Pharmacy and Food Industry

**DOI:** 10.3390/plants13192724

**Published:** 2024-09-29

**Authors:** Desislava Popstoyanova, Anelia Gerasimova, Galia Gentscheva, Stoyanka Nikolova, Anna Gavrilova, Krastena Nikolova

**Affiliations:** 1 Department of Physics and Biophysics, Faculty of Pharmacy, Medical University of Varna, 9002 Varna, Bulgaria; desislava.popstoyanova@mu-varna.bg; 2 Department of Chemistry, Faculty of Pharmacy, Medical University of Varna, 9000 Varna, Bulgaria; anelia.gerasimova@mu-varna.bg; 3 Department of Chemistry and Biochemistry, Medical University-Pleven, 5800 Pleven, Bulgaria; 4 Department of Organic Chemistry, Faculty of Chemistry, University of Plovdiv Paisii Hilendarski, 4000 Plovdiv, Bulgaria; tanya@uni-plovdiv.bg; 5 Department of Pharmaceutical Chemistry and Pharmacognosy, Medical University-Pleven, 5800 Pleven, Bulgaria; anna.gavrilova@mu-pleven.bg

**Keywords:** *Ziziphus jujuba*, chemical composition, pharmacological application, ecology

## Abstract

*Ziziphus jujuba* has been used since ancient times in traditional Eastern medicine. It is widely cultivated in numerous countries between the tropical and temperate climatic zones due to its high ecological plasticity and resilience to adverse weather. The different classes of chemical compounds contained in the plant are the reason for its medicinal properties. Research shows that every part of *Ziziphus jujuba*, the leaves, fruits and seeds, demonstrate therapeutic properties. This review focuses on the chemical composition in order to establish the relationship between the plant and its clinical use. Various biological effects are summarized and discussed: anticancer, anti-inflammatory, immunostimulating, antioxidant, hepatoprotective, gastrointestinal, etc. Apart from medicinal uses, the fruits of *Ziziphus jujuba* are edible and used in fresh and dried form. This literature review reveals possible medical applications of *Ziziphus jujuba* and its great potential for improving the diet of people in areas where the plant is abundant.

## 1. Introduction

Since ancient times, plants have been used not only for food but also for the treatment of people and animals. Over time, many plant species have spread far from their original place of origin. Such is the case with the plant known as *Ziziphus jujuba* Mill, commonly known as red date. It belongs to the Rhamnaceae family [1,2,3,4]. The species originates from China and has over 4000 years of history. It is considered one of the most valuable plants in Chinese traditional medicine [3,4,5,6]. The first scientific description of the species was made in 1753 by Carolus Linnaeus, misclassifying it as *Rhamnus ziziphus*. But in 1768, the error was corrected and the plant was designated as a new species [7]. There are around 40 jujube species known around the world [1]. Some of the most popular are *Ziziphus jujuba* (China), *Ziziphus mauritiana* Lam., *Ziziphus nummularia* (Burm.f.), *Ziziphus celata*, *Ziziphus lotus* (L.) Lam. (India), *Ziziphus obtusifolia* (US, Mexico), *Ziziphus parryi* Torr. (US, Mexico), *Ziziphus reticulata* (Vahl) DC., *Ziziphus rignonii* (Indonesia), and *Ziziphus taylorii* (Britton) [8].

In China, India, and other South Asian countries, the plant is considered a superfood. Moreover, it plays a role in local traditions and is among the five most valuable fruits according to the Huangdi Neijing (475–221 BC). There are four medicinally important species—*Ziziphus jujuba*, *Ziziphus glaberata*, *Ziziphus mauritiana*, and *Ziziphus rugosa* [9]. The first presents great interest to researchers as it is the fastest-growing of the four.

*Ziziphus jujuba (Z. jujuba)* has spread in different countries around the world. It could be found in many Asian countries: Afghanistan, Armenia, Azerbaijan, Bangladesh, Burma, China, India, Iraq, Iran, Israel, Japan, Kyrgyzstan, Lebanon, Malaysia, Mongolia, Pakistan, Palestine, South Korea, Syria, Thailand, Turkey, Turkmenistan, and Uzbekistan. In Europe, the plant grows in all southern countries, France, Germany, the Czech Republic, England, and Ukraine. In Africa, *Ziziphus jujuba* is found in Egypt, Tanzania, and Tunisia, as well as in Canada, the USA, Australia, and New Zealand [6]. However, China is still the only one that exports jujube as it grows 1.5 million hectares [10]. The annual sum of the dried plant has a value of around USD 5 million [11].

### Taxonometry

*Ziziphus jujuba* is classified as follows [8]:

*Domain: Eukaryota*;

*Kingdom: Plantae*;

*Subkingdom: Viridiplantae*;

*Phylum: Spermatophyta;*

*Subphylum: Angiospermae*;

*Superdivision: Embryophyta*;

*Division: Tracheophyta*;

*Subdivision: Spermatophytina*;

*Class: Magnoliopsida*;

*Superorder: Rosanae*;

*Order: Rosales*;

*Family: Rhamnaceae*;

*Genus: Ziziphus*;

*Species: Ziziphus jujuba*

A thorough description of the plant was made by Shahrajabian et al. [6]. The height of the plant is from 457 to 1070 cm on average and it spreads for 305 to 915 cm. The crown of the tree is oval, with irregularities in the silhouette and open density. The growth rate is classified as medium. The leaves have alternating arrangement, simple form, crenate or serrulate margins, ovate shape, and bowed venation. Their color is green in spring and summer and turns brown before shedding in autumn. The length reaches 10.5 cm. The plant blooms in spring with small yellow flowers that are hard to notice. The fruits are oval and 2.5 to 12.5 cm in length. Their color ranges from red to black. They are easy to spot on the tree, have fleshy texture, and attract animals. Stefania et al. [3] investigated the pathogen species found on the fruit after harvest. Most of them belonged to the *Rhizopus* spp. Others were *Alternaria* genus, *Monilinia* spp., *Fusarium* spp. The tree grows in full sun or partial shade. The plant can survive drought and lives in clay or sandy, well-drained soil [6,12]. All parts of the jujube have medicinal effects [13]. The literature includes reviews that examine the bioactivity of jujube extracts and the results of some in vitro and in vivo studies [14], antibacterial, phytotoxic, and hemagglutination activities [9], pharmacological effects and potential active ingredients [15], and the chemical components and biological activities of its fruits [16]. The purpose of this review is to summarize the chemical composition of *Ziziphus jujuba* Mill, its pharmacological and toxicological effects, its applications in the food industry, as well as other unconventional uses that have not been covered in other reviews but contribute to the understanding of its benefits.

## 2. Methodology

In the literature review, data and information regarding the botanical description of the species *Ziziphus jujuba*, its chemical composition and nutritional value, pharmacological action, and unconventional ecological applications have been collected, summarized, and analyzed. The articles used for *Z. jujuba* Mill. were sourced from Web of Science, Scopus, PubMed, ScienceDirect, SpringerLink, and Google Scholar. The search period covers articles published between 1976 and 2024. The following keywords were used: *Ziziphus jujuba* Mill., chemical composition, pharmacological application, ecological application, and botanical origin. About 200 literature sources were reviewed, of which approximately 132 were used in the review, while 68 were irrelevant as they dealt with other species of the studied plant.

## 3. Chemical Content

The chemical content of *Ziziphus jujuba* has been the objective of many studies. The richness in amino acids, such as tryptophan, is important for mental health and the optimal function of the brain [3]. The presence of many compounds such as tannin, saponins, flavonoids, terpenoids, phenolic compounds, alkaloids, amino acids, sugar, protein, fats, calcium, potassium, phosphate, and iron have been reported in the literature [1,17,18,19]. A thorough review of the various compounds was conducted by Aafi et al. [20], where they are sorted by class. The authors examined some of them in order to establish the relationship between the plant and its medicinal uses.

Flavonoids are a class of substances with some pronounced health benefits. The extraction method affects the total amount of flavonoids. The highest values were achieved with the extractant—chloroform—and the lowest were achieved with hexane in the leaves and methanol in the fruits [8].

Others types of substances that are found in the plant are quercetin glycosides (quercetin-3-glucoside, quercetin-3-rhamnoside), rutin (quercetin 3-O-rutinoside), quercetin 3-O-β-D-galactoside, kaempferol 3-O-robinobioside, quercetin 3-O-β-D-glucoside, kaempferol 3-rutinoside, 3′,5′-Di-C-β-D-glucosylphloretin, catechin, epicatechin, and procyanidin B2 [21,22,23,24].

Using FCR analysis, Hossain, M.A. determined the phenolic content of the plant, the best results being obtained with the ethyl acetate extract [8]. The results obtained suggest that the antioxidant effect of the plant can be explained by the content of flavonoids and polysaccharides [25,26,27]. Furthermore, the fruit contains vital vitamins in high concentrations—A, B1, B12, and C [17,27,28,29]. Data from the World Health Organization and Food and Agriculture Organization suggests that one fruit could be sufficient for the daily requirement of five different vitamins.

The polysaccharide content of *Ziziphus jujuba* also affects its uses. Jingya Ruan and others have conducted studies of this type of molecule in the plant. Forty-six types of polysaccharides are contained in the *Ziziphus jujuba* plant. Some of them have antioxidant, anti-fatigue, anti-inflammatory, liver protecting, and other healing properties. Such examples contain monosaccharides such as uronic acid, galactose, xylose, arabinose, galacturonic acid, etc. [30,31].

**Figure 1:**
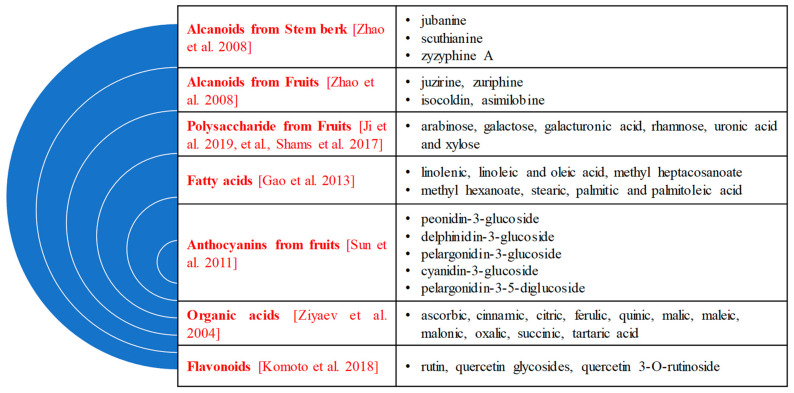
Main chemical compounds in different parts of *Z. jujuba* [17,21,30,31,32,33,34,35].

**Figure 2:**
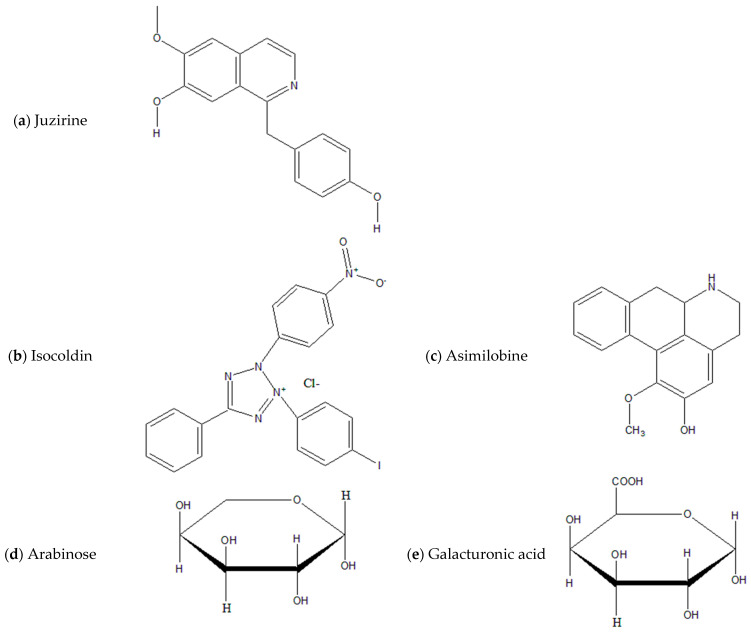

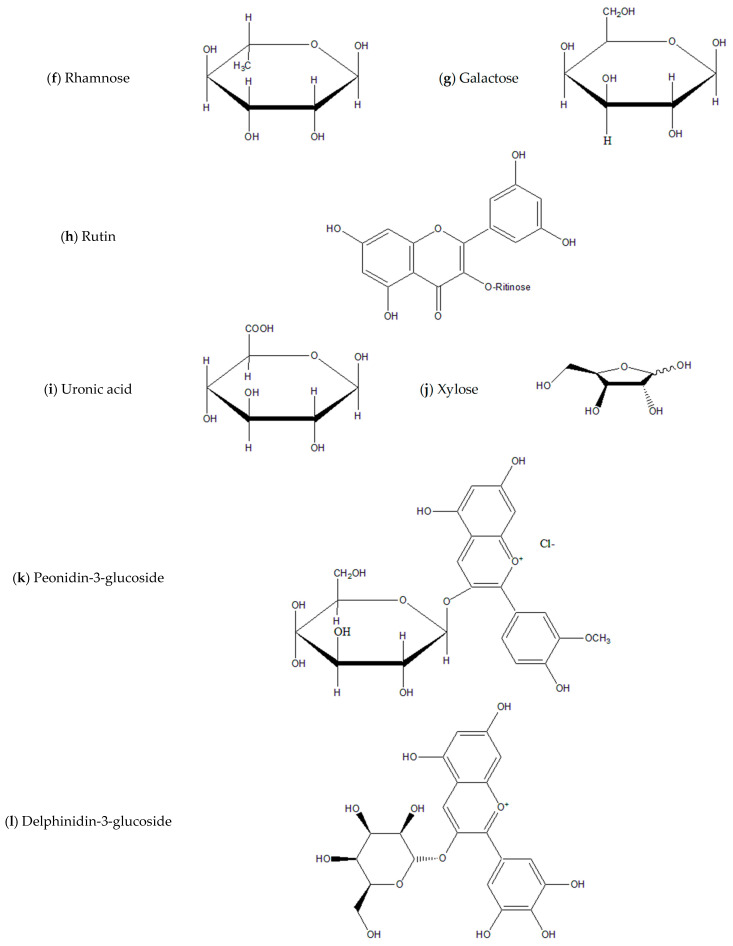

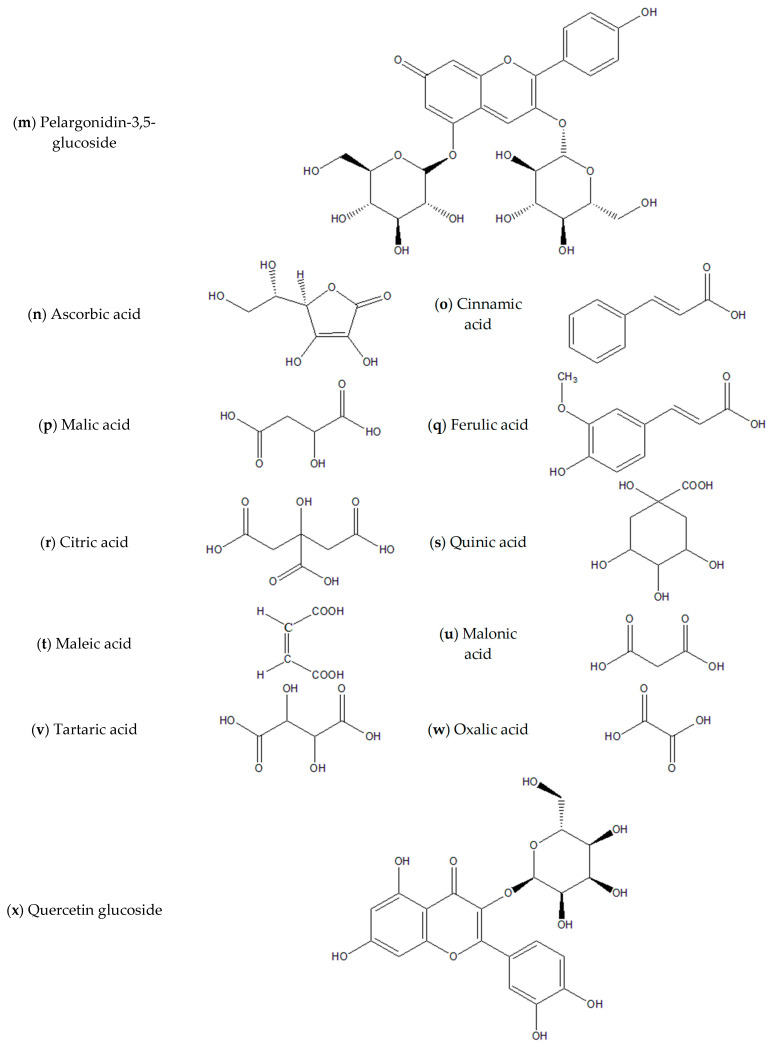
Structural formulas of the main chemical components in *Z. jujuba* applications in pharmacology.

The alkaloids in *Z. jujuba*, identified by Zhao et al., were asimilobine, isoboldine, juziphine, juzirine, and norisoboldine [31]. The chemical content of *Z. jujuba* is presented in Figure 1. The structure formulas of the main compounds are shown in Figure 2.

Table 1, Table 2, Table 3 and Table 4 present various chemical compounds and their concentrations reported for different countries around the world, indicating that climate and geographical region matter.

## 4. Pharmacological and Toxicological Effects

According to the literature, fruit and leaf extracts show pharmacological and toxicological effects depending on the polarity of the solvent [17,29,46,47,48]. The influence of jujube has been tested in vitro, however, there are few clinical trials to prove a real pharmacological application [20].

Its antioxidant activity has an important pharmacological application. It was determined by a modified radical scavenging method by several authors [1,2,49,50]. *Z. jujuba* exhibited reduction activity over iron and lipid peroxidation assays [20]. The authors [20] found that the highest antioxidant activity was shown by the fruit extracts with chloroform. The scavenging activity decreased with the polarity of the sample. Another paper determined that the peel had the highest antioxidant activity [51]. The reason for this activity is linked directly to the chemical content of the sample—uronic acid, flavonoids, and phenolic compounds in particular. The influence of uronic acid and how it affects the antioxidant potential of polysaccharides was studied by Li et al. [27]. Fruit quality varies depending on different growing conditions and harvesting methods. It is suggested that high-stress environments and higher altitudes increase the antioxidant activity [52]. Sun et al. [35] conducted a comprehensive evaluation of the total antioxidants and antioxidant activity in relation to environmental conditions by examining samples from ten different regions with varying latitudes and altitudes in China. They found a positive correlation between altitude and the levels of polyphenols, anthocyanins, saponins, flavonoids, and polysaccharides while observing a negative correlation with vitamin C. Annual rainfall had a strong negative impact on the content of flavonoids, polyphenols, saponins, polysaccharides, and carotenoids, but a positive effect on the content of vitamin C.

According to the results of experiments on rats, the polysaccharides reduced the trauma on the liver due to CCl_4_ poisoning. The levels of 3,4-methylenedioxyamphetamine (MDA) were also reduced, and the activity of the antioxidant proteins stayed within normal limits [9]. Another study suggests that the levels of antioxidant enzymes (superoxide dismutase (SOD) and catalase (CAT)) and glutathione (GSH) were increased, and the levels of aminotransferase enzymes (ALT and AST) and MDA were decreased. The histological activity was also improved [53,54,55]. Some neurodegenerative diseases, such as Parkinson’s and Alzheimer’s, are greatly influenced by oxidative stress as their progression is accelerated. Experiments with neuronal cell lines (PC12) investigating the protective properties of jujube water extract against tert-butyl hydroperoxide-induced oxidative damage have had positive results. The fraction further reduces the reactive oxygen species formed from the agent [56]. *Z. jujuba* was tested in vivo against ischemic damage in the gerbil hippocampus; the effect was attributed to the upregulation of superoxide dismutase and reduced lipid peroxidation [57].

Another useful property of *Z. jujuba* is its anti-inflammatory activity. It was observed in experiments with mice in acute and chronic cases. The inhibition was achieved by interaction and deactivation of COX, NO, and histamine. The most active compounds of the plant were the terpenoid acids [58].

Al-Saeedi et al. studied the antimicrobial activity of *Z. jujuba* by tests performed on Gram-positive and Gram-negative bacteria. The extracts of the plant showed potency against the tested strains. The inhibition depends on the ingredients of the drug solution. The authors found that the best results were obtained using the hexane extracts. This suggests that this type of extraction contains the biggest concentration of bioactive compounds. There is a difference between studies due to the extraction methods, bacterial stains, doses, etc. Other experiments showed antimicrobial activity against *Escherichia coli* and *Staphylococcus aureus* [59]. The ethyl acetate extract has antimicrobial effects against *Bacillus pumalis*, *Pseudomonas aeruginosa*, *Salmonella typhi*, and *Staphylococcus epidermidis*. For example, the n-hexane fraction of *Z. jujuba* is significantly active against *B. pumalis*, while an aqueous fraction is active against *P. aerugenoza* [9]. Snakin-Z is a peptide derived from *Z. jujuba* that showed antimicrobial effect on *E. coli*, *K. pneumonia*, *B. subtilis*, *S. aureus*, *Aspergillus niger*, *Candida albicans*, *Phomopsis azadirachtae*, *and Pythium ultimum* with higher activity on Gram-positive bacteria [60]. Betulinic acid extracted from the plant shows an activity against the influenza virus in experiments with mice [61].

The fruit of *Z. jujuba* expresses neuroprotective effects in in vitro experiments. Glucose-induced neurotoxicity is reduced in a model of diabetic neuropathy [62]. Taati et al. found that consumption of the fruit lowers ethanol-induced spatial memory loss and oxidative stress [63]. Moreover, some experiments in the literature suggest that water fruit extract is beneficial to neuron differentiation. The effect was observed in vitro and was modeled using PC12 cell lines. After 72 h, proliferation and stimulated expression of certain neurofilament proteins were induced [56]. Another important part of the nervous system is played by astrocytes. They produce neurotropic factors such as NT3, NT4/5, BDNF, and GDNF. Their upregulation is vital to the cell as it plays an important role throughout its whole living cycle. There have been some in vitro experiments on the effect of *Z. jujuba* on astrocyte cellular lines. The result was a dose-dependent elevated expression of the above-mentioned factors while the morphology of the cells remained unaffected [64]. Jujube was also observed to improve the signaling pathway cAMP-PKA-CREB, which is connected to neuron differentiation. This effect and the improvement in the expression of neurotrophic factors can both be inhibited by the addition of H89 [64,65].

One of the promising substances that could be used against Alzheimer’s disease is oleamide. It has been extracted from jujube fruits and could be used for treatment [66]. Snakin-Z has the properties to treat this disease. Its neurobiological activity has been studied, and in addition, it also has antioxidant and cholinesterase inhibition activity [67].

Another application of *Z. jujuba* has been suggested to be antiepileptic activity. The hydroalcoholic extract from the fruit reduced the seizures expressed by rats. Anticonvulsant activity was observed with both types of seizures—tonic-clonic and tonic hindlimb extension. The fruit also increased the learning ability and memory of mice [68]. The synergy with antiepileptic drugs on the market has also been examined. The concentrations of the drugs were not changed by the addition of the plant extract. In two cases—with phenobarbital and phenytoin—*Z. jujuba* improved the effect. No change was observed with simultaneous administration of carbamazepine and the fruit [69].

*Ziziphus jujuba* is used in traditional Chinese medicine to battle insomnia [70]. This was confirmed in experiments with mice as jujube increased sleep time and decreased the movement of the subjects [71]. Other research on jujube seeds reports similar results in both mice and rats [72]. An extract of flavonoids and saponins from the seed decreases coordinated movement and prolongs sleep [73]. Another effect of jujube is the improvement in learning ability and memory, as mentioned above. An increase in estrogen in the blood and nitric oxide and acetylcholine in the brain was probably the cause in the ovariectomized rat model [74]. Enhancement in memory from the intake of *Z. jujuba* extract was reported in alcohol-induced disorders, and improvement in learning ability was observed [75].

The polyphenols in jujube can have cardioprotective activity. The results from rat experiments show reduced MDA activity and inhibited expression of ST-segment in ECG. They enhance SOD, GPx in the hearts of the tested animals. The myofibrillar degeneration of the left ventricle is reduced [76].

The hydroalcoholic extract from the jujube fruit was tested using two intake methods—topical gel and oral consumption—against ulcerative colitis in rats. Both were found to be effective; however, the gel had more potency—around 40%. The colon tissue histopathology was enhanced, and the levels of interleukin-1β (IL-1β), myeloperoxidase, and GPx were reduced [77]. There was a clinical trial on the effect of jujube against constipation. The test group was 50 patients, and the period was 12 weeks. The treatment had a significant effect on the group that received jujube compared to the placebo group. This method positively influenced the cases of chronic idiopathic constipation, bloating, and abdominal pain [78]. *Ziziphus jujuba* also reduces inflammation and progression of colon cancer [79].

In total, 70% of ethanolic extracts of *Z. jujuba* were evaluated using Caco2 cells and a mouse model and demonstrated their efficacy in IBD (inflammatory bowel disease) [80].

Due to its content of flavonoids and steroidal saponins, *Z. jujuba* also has a protective activity against allergies and asthma. Its effect has different mechanisms of positive interaction, including stabilization of mast cells, immunomodulation, antihistamine effect, etc. [81,82].

Another application is in the healing of second-degree burn wounds. The fruit extracts significantly accelerate the process [83].

*Ziziphus jujuba* is also used in ginseng paste as an adjuvant to improve the treatment of hyperuricemia. This is a disease caused by the overproduction of uric acid. It was shown that the ginseng–jujube pair could have a positive influence on the illness through interaction with CCL2, TNF-α, IL-1β, and VEGFA. The effect of hydroalcoholic extracts of *Z. jujuba* leaves on the prevention/treatment of kidney stones was evaluated and found to reduce the formation of calcium oxalate crystals and the effective dose was 500 mg/kg [84].

A brine shrimp lethality assay was used by Al-Saeedi et al. to determine the cytotoxicity of *Z. jujuba* extracts. It was shown that butanol leaf extract had the highest toxicity. This characteristic depends on the ingredients of the sample. These results were confirmed by Hoshyar and Alhakmani [44,85].

Moreover, the fruits of *Ziziphus jujuba* have an immunostimulatory effect. One of the water-soluble fractions, ZSP3, which has a lot of pectin, shows a potential immunological response with 49% esterification [27]. The effect of *Ziziphus jujuba* fruit extract on the non-specific immune parameters of skin mucus and the mRNA levels of immune-related genes in the skin of small carp was investigated. Feeding of 0.5 and 1% *Ziziphus jujuba* fruit extract was found to significantly increase the protease activity of skin mucus, and gene expression studies in skin showed a significant increase in Il1b expression. [86]. This part of the plant is used in aesthetics and medical cosmetics [87]. The healing properties of *Ziziphus jujuba* have also been tested on wounds. Breastfeeding is negatively affected by nipple fissure pain. Several lactating women have tested lotion with an extract of the plant’s fruit and reported that the pain subsided between one and two weeks after using it [88].

**Figure 3:**
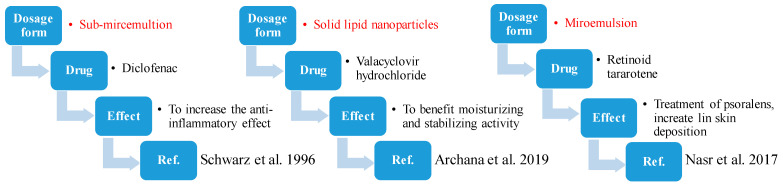
Pharmaceutical forms containing jujube oil [91,92,93].

According to the literature, essential oil extracted from *Ziziphus jujuba* enhances skin elasticity and enables bigger elastic deformations. Miwa suggested that it could be used to improve the treatment of acne and psoriasis [89]. Later, Pazyar noted the potential of the oil for acne treatment due to its positive keratoplastic effect [82]. Furthermore, some pharmaceutical advancements have improved the penetration capability of drugs or the delivery efficiency to the target area due to the inclusion of the jujube’s oil. An example is the enhancement of the flow of the medication olanzapine through the skin in transdermal patches. It is often used in the emulsification of alginate beads with hydrogels for the production of vaccines in order to improve their stability [90]. For instance, the vaccine Calmette-Guérin is stable at room temperature for as long as a year after dehydration using freezing of such beads [91]. The essential oil of jujube is included in a lot of pharmaceutical products. In Figure 3, an example of three of them is presented.

Lee et al. [94] found that *Zizyphus jujuba* var. inermis (Korea) essential oil is a rich source of 3-pentadecylcatechol, ρ-menth3-ene, γ-bisabolene, and vomifoliol, as well as β-sitosterol, stigmasterol, stigmasta-5,23-dien-3β-ol, stigmast-4-en-3-one, lupeol, betulinic acid and its derivatives, alphitolic acid and its derivatives, zizyberanalic acid, ceanothic acid, oleanolic acid, and maslinic acid and its derivatives.

Elaloui et al. [95] identified thirteen fatty acids from the pulps’ essential oil of four Tunisian *Ziziphus jujuba* ecotypes (Sfax, Choutrana, Mahres, and Mandia). The authors found that Mahres and Choutrana were the richest sources of oleic acid compared to the other ecotypes. The Sfax ecotype’s most significant chemical compound was palmitic acid. For each ecotype, the percentage of unsaturated fatty acids ranged from 62.63% to 72.40% of total fatty acids. The unsaturated/saturated (U/S) ratio therefore ranged from 1.68 to 2.37. Beta-sitosterol and stigmasterol were found to be the major sterols. 

Out of the 20 compounds found in the essential oil from *Ziziphus jujuba* seeds [96], the most abundant ones were 13-Heptadecyn-1-ol (12.95%), 7-Ethyl-4-decen-6-one (9.73%), Lineoleoyl chloride (8.54%), Linoleic acid (6.37%), 2,5-Octadecadiynoic acid, methyl ester (5.57%), and Palatinol A (4.81%).

## 5. Applications in Foods

The jujube’s fruits have a short shelf-life—around 10 days in fresh form. The pulp of the jujube is usually eaten fresh, but the fruit could also be dried and included in other recipes [97]. The remarkable healing potential of *Ziziphus jujuba* renders it a staple in traditional cuisine and an increasingly popular choice as a functional food. Its incorporation into culinary practices not only enhances flavor profiles but also infuses dishes with a myriad of health benefits, contributing to its growing prominence as a sought-after ingredient in both traditional and modern dietary contexts [98]. It can be added to improve the taste or to enhance the nutritional value. Rashwan et al. reviewed the content of more than 30 food products incorporating jujube. Some examples are tea, cake, yogurt, bread, etc. A couple of the jujube’s products have been investigated further in order to evaluate their medicinal properties. The powdered form exhibited high levels of phenolic compounds. Moreover, it expressed antimicrobial, antioxidant, and anticancer activity. This form has already found its application in the treatment of dyslipidemia and insulin resistance [92,93,94,95,96,97,98,99,100,101,102]. The jujube vinegar expressed anticancer properties and a positive effect on the gastrointestinal system. It is used in insomnia treatment and normalizes blood pressure. The vinegar also contains vitamin C [103,104,105]. Another product—jujube fermented juice—has the potential to be used in non-dairy probiotic food. It has an abundance of phenolic compounds. The same characteristics are applicable to jujube wine [105,106,107,108,109,110]. Fresh juice is rich in Vitamin C and has antioxidant properties [104,105,111].

In order to avoid fast spoilage, food processing is used to prevent bacteria, yeast, and fungi from developing on the product. This improves the storage time of the culinary item. On the other hand, the process has to be optimized to ensure that the item retains its valuable qualities. According to Zozio et al., jujubes’ shelf life is around 10 days in uncontrolled conditions [112]. Sensory evaluation and chemical analysis were performed by Shin and co-authors. It was concluded that powder, dried fruits, extracts, juices, and jam showed the greatest preserving potential [113]. A solely sensory evaluation of different products after various preservation methods (cloying with honey, vinegar infusion, compote, and drying) was made by Krška [97]. On their scale, the compote was ranked the highest. The jam, jelly, and pickled jujube have been tested for their physicochemical and sensory characteristics by Uddin and Hussein in 2012. The nutrition in these forms was retained; they complied with food standards and were satisfactory [114].

Drying food is considered a suitable preservation process that maintains the flavor, aroma, and color of the *Z. jujuba* fruits. This is a reason to investigate some innovative techniques further. Some examples use infrared and microwave radiation, vacuum, freezing, or a combination of these methods. They increase the speed of the process while sustaining the qualities [115,116,117,118].

Juices are easy to consume and are a suitable addition to food supplements. The extraction method can vary in parameters. A study showed that when using hot water, the optimal conditions are 70–80 °C and 40 min in a fruit-to-water ratio of 1:7. Enhanced effect is achieved by following this procedure by pectinase treatment [98].

Another common beverage is *Z. jujuba* wine. However, research on its ideal preparation conditions is scarce compared to the above-mentioned examples. Liu et al. and Zhao et al. concluded that suitable conditions are 18% sugar content, pH = 4.0, 24 °C, and 12.5% vol. alcohol in the wine produced [119]. Jujube’s brandy (50% vol.) is a popular drink in China. The evaluation of such beverages is based on the content of volatile organic components (VOC) as they are major components of the aroma. This characteristic can be altered with variations of the processing methods. For example, thermal treatment increases VOC while decreasing phenolic and flavonoid compounds by around 20%. Some of the contents of jujube wine include 16 amino acids and seven organic acids. Their quantity was increased with the process of fermentation, which indicates they improved the taste [98]. Jujube’s vinegar is another available product. It has high nutritional value as well as increased content of antioxidant components. This classifies it as functional vinegar [105].

Candied *Z. jujuba* is one of the more well-known products in China. This form has a longer storage time and could be prepared with different sweeteners in order to lower the sugar content [98]. Different methods vary in the retention of valuable substances such as vitamin C. Zhao et al. investigated different recipes that are used to prepare jam and jelly from jujube’s fruit [118,119]. Pectin obtained from *Z. jujuba* at different ripeness was studied, and it was found that it can be used to increase the amount of vitamin C in kefir [120].

Some artificial food additives can be substituted with jujube to decrease health hazards. Some examples are the addition of bread or meat to improve the preparation process [121].

Since quinoa, although a complete food, due to its bad taste, is not a good option for direct consumption (cake), this shortcoming was overcome by adding jujube fruit powder [122].

In Chinese alternative medicine, there is an increasing focus on functional foods that meet the needs of the population. Bae et al. studied yogurt with added fermented seeds of *Ziziphus jujuba* using *Lactobacillus brevis* L-32 [123]. The authors reported that the resulting fermented beverage was high in γ-aminobutyric acid (GABA), which promotes sleep. The addition of pulp from *Ziziphus jujuba* fruits improves the taste of goat yogurt [124]. Furthermore, the addition of polysaccharides extracted from the pulp of *Ziziphus jujuba* fruits enhances the density and firmness of the casein matrix structure in goat cheese [125].

## 6. Other Applications Related to Zero-Waste Technologies and Ecology

Recently, special attention has been paid to the waste-free use of plant resources, from composting to the production of non-conventional adsorbents.

The pulp of *Ziziphus jujuba* fruits can be used to extract condensed tannins [126]. Research by the author team has shown that these substances effectively inhibit the monophenolase and diphenolase activities of tyrosinase. Therefore, *Ziziphus jujuba* pulp is a natural tyrosinase inhibitor with potential applications in food preservation and skin-whitening cosmetics.

Steam-pyrolyzed at 1000 °C, *Ziziphus jujuba* seeds have been used to produce granular activated carbon with twice the adsorption capacity of commercially available options [127]. When the surface of the plant seeds is modified with ultrasound and sulfuric acid, they become suitable for the absorption of toxic metals such as cadmium, copper, zinc, lead, nickel, and others [128].

The chemical modification of *Ziziphus jujuba* stones (ZJS) with ortho-phosphoric acid (ZJS-H_3_PO_4_) was found to have an adsorption capacity of 179.83 mg g^−1^ of methylene blue, and they could be used as efficient adsorbents for organic dyes [129]. Jujube kernel fibers (JKF) can serve as a renewable and environmentally friendly wastewater adsorbent after modification of their microstructure. JKF treated with mixed enzymatic hydrolysis and acetylation can adsorb oil, while JKF treated with mixed enzymatic hydrolysis and carboxymethylation have high adsorption ability for heavy metals [130]. Gavade et al. reported a one-step biogenic synthesis of silver nanoparticles (AgNPs) by using *Ziziphus jujuba* leaf extract as a reducing and stabilizing agent. These particles can be used in environmental applications to reduce the anthropogenic pollutant 4-nitrophenol (4-NP) and methylene blue (MB), and they also show good antimicrobial activity against *Escherichia coli* [131]. A study investigated the mechanical properties of hybrid composites prepared from *Holoptelea integrifolia* bark fiber (HIBF) reinforced with *Ziziphus jujuba* seed particles (ZJSP) for bio-based epoxy resin (BBER) [132]. A highly porous, economical, and sustainable carbon electrode based on a waste material (*Ziziphus jujuba* seeds) has been developed, which can be useful for energy storage [133].

Due to their high energy value, jujube fruits have shown a positive effect when used as an alternative additive in diets for poultry in egg production, which further helped reduce feed [134].

## 7. Conclusions

In conclusion, this review presents a description of the main chemical compounds found in the plant *Z. jujuba* with pharmacological importance. Attention has been given to its application in the food industry. Our evaluation shows that all its parts—leaves, fruits, and seeds—have medicinal potential. However, further clinical studies of the effects of various *Z. jujuba* extracts are lacking for most reported uses. It has been noted that the fruit retains some of its valuable constituents after drying, and these properties can be used in herbal mixtures. In addition, *Z. jujuba* has other applications: as a sorbent, animal feed, and a composite material.

## Figures and Tables

**Table 1:** Sugar content of fruits from *Ziziphus jujuba* from different parts of the world.

Country	Type of Sugar	Value	Reference
China	Fructose	(3.24–8.66), g/100 g fw	[36]
	Glucose	(2.97–8.44), g/100 g fw	[36]
	Sucrose	(16.39–25.8), g/100 g fw	[36]
Spain	Fructose	(4.8–5.7), g/100 mL	[37]
	Glucose	(3.0–3.9), g/100 mL	[37]
	Sucrose	(1.6–9.4), g/100 mL	[37]
Italy	Fructose	* (0.43–0.63), g/100 g fw	[38]
		** 0.81, g/100 g fw	
	Glucose	* (2.35–3.35), g/100 g fw	[38]
		** 3.28, g/100 g fw	
	Sucrose	* (0.97–1.38), g/100 g fw	[38]
		** 0.22, g/100 g fw	

* Organic Crop System; ** Wild System.

**Table 2:** Nutritional composition of fruits from *Ziziphus jujuba* from different parts of the world.

Type of Nutrient	Country	Value	Reference
Lipids	Senegal	(1.3–2.5), g/100 g	[39]
	Slovenia	2.5 g/100 g	[40]
	Iran	(0.40–0.72), g/100 g	[41]
	Turkey	(0.05–0.1) g/100 g	[42]
	Italy	(0.02–0.04), g/100 g *	[38]
		0.03 g/100 g/100 g **	
Proteins	Iran	(3.24–3.92), g/100 g	[41]
	China	(1.87–3.97), g/100 g	[36]
	Senegal	(1.7–6.9), g/100 g	[39]
	Italy	(0.29–0.40), g/100 g *	[38]
		0.26 g/100 g **	
Carbohydrates	Italy	* (21.55–31.74), g/100 g fw	[38]
		** 24.79, g/100 g fw	
		** 0.22, g/100 g fw	

* Organic Crop System; ** Wild System.

**Table 3:** Elements composition of fruits from *Ziziphus jujuba* from different parts of the world.

Type of Nutrient	Country	Value	Reference
Calcium	Senegal	(0.76–4.88), g/kg	[39]
	Slovenia	1.77 g/kg	[40]
	Spain	(0.23–0.72), g/kg	[37]
	Italy	(0.056–0.27), g/kg *	[38]
		0.065 g/ kg **	
Potassium	Italy	(0.90–2.26), g/kg *	[38]
		1.20 g/ kg **	
	China	(2.066–7.183) g/kg	[43]
	Slovenia	8.29, g/kg	[40]
	Spain	(11.9–13.8), g/100 g *	[37]
Sodium	Italy	(0.0392–0.0962), g/kg *	[38]
		0.043 g/ kg **	
	China	(0.00378–0.375), g/kg	[43]
	Slovenia	1.77, g/kg	[40]
	Spain	(0.11–0.43), g/kg *	[37]
Magnesium	China	(0.147–0.400), g/kg	[43]
	Senegal	(0.21–1.37), g/ kg	[39]
	Italy	(0.0811–0.197), g/ k *	[40]
		0.0748 g/kg **	

* Organic Crop System; ** Wild System.

**Table 4:** Bioactive components of the jujube fruits.

Country	Type of Bioactive Components	Value	Reference
Spain	Citric acid	(0.3–0.77), g/100 mL	[37]
	Ascorbic acid	(0.41–0.64), g/100 mL	[37]
	Carotenoids	(0.22–0.39), mg/100 g fw	[44]
	Total Chlorophyl	(0.29–0.51), mg/100 g fw	[44]
	Total antioxidant activity ABTS	(31.30–51.07), mM Trolox/kg fw	[44]
	Total antioxidant activity DPPH	(199.81–322.15), mM Trolox/kg fw	[44]
	Total antioxidant activity FRAP	(52.21–109.87), mM Trolox/kg fw	[44]
Ukraine	Carotenoids	(1.53–14.31), μg/g	[45]
	Total Polyphenol content	(8.76–21.61), mg GAE/g	[45]
	Total Flavonoids content	(1.49–11.59), μg QE/g	[45]
	Total antioxidant activity DPPH	(11.18–16.82), mg TEAC/g	[45]
Turkey	Beta-carotene	(7.00–35.00), μg/100 g	[42]
Italy	Total antioxidant capacity	* (213.74–241.31), μM ET/100 g fw	[38]
		** 243.14, μM ET/100 g fw	
	Total Polyphenols	* (480,83–630,81), mg EGA/100 g fw	[38]
		** 520.71, mg EGA/100 g fw	
	Ascorbic acid	* (288.10–441.13), mg/100 g fw	[38]
		** 303.06, mg/100 g fw	
		** 0.22, g/100 g fw	

* Organic Crop System; ** Wild System.

## Data Availability

The data presented in this study are available on request from the corresponding authors.
